# The Role of CXCR3 and Its Chemokine Ligands in Skin Disease and Cancer

**DOI:** 10.3389/fmed.2018.00271

**Published:** 2018-09-25

**Authors:** Paula T. Kuo, Zhen Zeng, Nazhifah Salim, Stephen Mattarollo, James W. Wells, Graham R. Leggatt

**Affiliations:** Diamantina Institute, Translational Research Institute, University of Queensland, Brisbane, QLD, Australia

**Keywords:** CXCR3, skin cancer, CXCL9/10/11, melanoma, squamous cell carcinoma

## Abstract

Chemokines and their receptors play an important role in the recruitment, activation and differentiation of immune cells. The chemokine receptor, CXCR3, and its ligands, CXCL9, CXCL10, and CXCL11 are key immune chemoattractants during interferon-induced inflammatory responses. Inflammation of the skin resulting from infections or autoimmune disease drives expression of CXCL9/10/11 and the subsequent recruitment of effector, CXCR3^+^ T cells from the circulation. The relative contributions of the different CXCR3 chemokines and the three variant isoforms of CXCR3 (CXCR3A, CXCR3B, CXCR3alt) to the inflammatory process in human skin requires further investigation. In skin cancers, the CXCR3 receptor can play a dual role whereby expression on tumor cells can lead to cancer metastasis to systemic sites while receptor expression on immune cells can frequently promote anti-tumor immune responses. This review will discuss the biology of CXCR3 and its associated ligands with particular emphasis on the skin during inflammation and carcinogenesis.

## Introduction

Chemokines are secreted, chemotactic cytokines ranging from 8 to 14 kDa in size and are classified into four groups based on the position of the conserved cysteine residues: CXC, CC, C and CX_3_C (1). Chemokines typically interact with chemokine receptors, which are seven-transmembrane proteins of the G protein-coupled receptor (GPCR) superfamily (2). Through these interactions, chemokines deliver diverse signal transmission to direct leukocyte migration, inflammation and differentiation (3, 4). The importance of chemokines and chemokine receptors in regulating inflammatory processes is underpinned by their involvement in the pathogenesis of several autoimmune diseases, including rheumatoid arthritis (5, 6), inflammatory bowel disease (7), type I diabetes (8, 9), and psoriasis (10, 11).

In some cases, multiple chemokines can interact with the same chemokine receptor resulting in functional redundancy (12, 13). However, some receptors, such as CXCR4, CXCR5, CXCR6, CCR6, CCR9, and CX_3_CR1 only interact with one known ligand (1). Selective ligand binding directs tissue specific and cell type specific lymphocyte recruitment. For example, CCR9 plays critical role in recruiting lymphocytes to the gut (7) while CCR6 drives Th17 homing to the site of inflammation (14).

More recently, chemokines have been observed to have functions other than simply being leukocyte attractants. Karin et al. termed chemokines that are able to alter the biological functions of recruited leukocytes as “driver chemokines” (15). For example, CXCL12 polarizes CXCR4^+^ macrophages into IL-10-secreting M2-like macrophages (16) and CXCR4^+^CD4^+^ T cells into IL-10-producing regulatory T cells (T_r_1) that suppress experimental autoimmune encephalomyelitis (EAE) (17). In addition to inducing immunosuppressive cell types, chemokine signaling can also influence effector T cell polarization. One example is the CXCR3 receptor which can help differentiate naïve T cells into Th1 effector T cells (18).

### CXCR3 and chemokine ligands

CXCR3 (GPR9/CD183) is an interferon-inducible chemokine receptor expressed on various cell types, but preferentially monocytes, Th1 T cells, CD8 T cells, NKT cells, NK cells, dendritic cells, and some cancer cells (19–21). Homeostatic proliferation of T cells in immune depleted individuals can also lead to an enrichment of CXCR3^+^ T cells (22). The CXCR3 receptor reacts with three interferon-inducible chemokines: CXCL9 (MIG), CXCL10 (IP-10) and CXCL11 (I-TAC/IP-9) in addition to CXCL4. Mice have a single isoform of the CXCR3 receptor while three isoforms, CXCR3A, CXCR3B, and CXCR3-alt, exist in humans with distinct roles in cell biology and tumorigenesis (23). CXCR3A plays a traditional CXCR3 role comprising chemotaxis and cell proliferation via calcium mobilization in immune responses induced by IFN-γ (24). With an extension at the N terminus of 52 amino-acids, CXCR3B is an alternatively spliced form of the CXCR3 receptor capable of inhibiting the growth of primary endothelial cell lines (25). CXCR3-B has also been shown to be expressed on endothelial cells within tumors and therefore may be involved in inhibiting angiogenesis through CXCL9/10/11 or CXCL4 signaling (25). The CXCR3-alt receptor results from alternative splicing generated by exon skipping (26). This leads to a truncation at the C-terminus of the protein allowing for binding and mediating the function of CXCL11. Each of these variant CXCR3 receptors can activate different intracellular signaling pathways suggesting that they have non-redundant roles in the immune response (27). A more complete understanding of the regulation and functional activity of these variant receptors during tumorigenesis should aid the design of therapeutic drugs which target the human CXCR3 receptor.

The CXCR3 receptor has become an important marker of a Th1 dominated T cell response. Co-expression of CXCR3 and CCR5 mark Th1 subsets (28), while CCR3 and CCR4 are preferentially expressed on Th2 subsets (29). Interestingly, CXCL11, and to a lesser extent CXCL9/10, is capable of binding and antagonizing the CCR3 receptor thus reducing the Th2 response (30). Xanthou et al. also demonstrated that CXCR3 can bind and sequester the CCR3 ligand, CCL11, again reducing the migration of CD4 Th2 cells. Consequently, CXCR3 and its ligands downregulate Th2 responses while promoting Th1 cell migration. In this context, CXCR3 functions include: (i) the recruitment of activated Th1 cells to inflamed tissues (31–33), (ii) the regulation of skin-homing autoreactive CD8^+^ T cells in graft-versus-host-disease (GVHD) (34), and (iii) the rapid recruitment of NK cells to antigen-stimulated lymph nodes and the facilitation of Th1 subset priming (35). Transcription factor T-bet, the master regulator controlling Th1 and CTL polarization, is also a direct trans-activator of CXCR3 expression (36). While T-bet upregulation of CXCR3 promotes the migration of Th1 effector cells to inflammatory sites, FoxP3^+^ regulatory T cells under the influence of IFN-γ can also induce T-bet and subsequently CXCR3 leading to the recruitment of these suppressive T cells into inflammatory sites (37, 38). Ultimately, the timing and number of recruited effector and suppressor cells will dictate the outcome of the immune response.

The key chemokine ligands of CXCR3 (CXCL9, CXCL10, CXCL11) have limited expression under homeostatic conditions but are rapidly up-regulated by cytokine stimulation. While CXCL9 is mostly induced by IFN-γ, CXCL10, and CXCL11 can be induced by both IFN-γ and type I interferons (21). Given the association between the CXCR3 system and inflammation, it is perhaps not surprising that CXCR3 and its ligands also play a role in a variety of autoimmune diseases (39–42). In response to IFN-γ, and synergistically enhanced by TNF-α, many cell types can secrete CXCL9/10/11 including endotheliocytes, fibroblasts, monocytes, and also cancer cells (21). There are two distinct groups of CXC chemokines: one with an ELR (Glu-Leu-Arg) amino acid motif and the other without. Those with the ELR motif can promote angiogenesis while ELR-negative chemokines principally promote lymphocytes migration and repress angiogenesis. CXCL9/10/11 lack the ELR motif thus attenuating angiogenesis to negatively impact on tumor growth (43, 44). Interestingly, CXCR3 and its ligands can also be responsible for tumor growth and metastasis in situations where the tumor cells express the CXCR3 receptor. In a study of CXCR3-expressing colorectal cell lines, metastasis to the liver and lung could be prevented with a small molecule inhibitor of CXCR3, AMG487 (45). In a nude mouse, knock down of human CXCR3A within gastric tumor cells led to reduced metastasis and tumor cell growth *in vivo* suggesting this receptor variant as the dominant mediator of metastasis in this model (46). Together, these observations suggest that CXCR3 receptor isoform expression and distribution throughout the tumor microenvironment, including on the tumor cells themselves, are important considerations when designing therapeutics that target the human CXCR3 receptor.

CXCL9, 10, and 11 have different binding affinity with CXCR3. Cole et al. showed that human CXCL11 binds to CXCR3 with the highest affinity followed by CXCL10 and CXCL9, although binding to CXCR3 receptor variants was not analyzed (47). This raises the question of whether CXCR3 ligands are redundant or compete during immune responses. The redundancy of CXCL9 and 10 has been demonstrated in a murine model of obliterative bronchiolitis (48). In this study the authors demonstrated that blockade of CXCR3 reduced airway obliteration while single deletion of either CXCL9 or CXCL10 had no effect. However, while CXCL9 and CXCL10 can drive Th1 responses, CXCL11 interaction with CXCR3 can selectively induce regulatory T cells (49, 50). CXCR3 ligands have also been shown to have cooperative effects. For example, murine CXCL9 and 10 cooperatively induce the recruitment of NK cells and CTLs to the spinal cord during herpes simplex virus-2 infection (51). In some cases, CXCR3 ligands can counteract one another. This is seen in a murine MHC-mismatched cardiac transplantation model, where CXCL9 and CXCL10 showed antagonistic effects toward the priming of donor-reactive T cells (52). CXCL9 deficiency decreased the frequency of donor-reactive IFN-γ-producing CD8 T cells, while deficiency of CXCL10 increased the frequency of CD8 T cells in a CXCL9 dependent manner (52). In summary, the interaction of CXCR3 and its ligands is complex and the outcomes will likely be controlled by spatial and temporal patterns of expression that could well be unique to each tissue including the skin.

As post-transcriptional regulators of target genes, multiple microRNAs (miRNAs or miRs) have been reported to regulate CXCR3 ligands. Downregulation of miR-21 in a breast cancer cell line raised secretion of CXCL10, resulting in enhanced recruitment of lymphocytes (53). Interestingly, miR-21 has been shown to be upregulated in cutaneous SCC suggesting that it may reduce CXCL10 recruitment of lymphocytes (54). Similarly, increasing the expression of miR-15a in PBMC results in decreased CXCL10 production (55). In human mesangial cells treated with IFN-γ and TNF-α, the expression of miR-155 was increased resulting in down regulation of CXCL10 while in the inflammatory skin setting of vulvar lichen sclerosus and lichen planus, miR-155 was significantly upregulated but the functional impact of this expression was not fully investigated (56, 57). The expression of CXCL9/10 from psoriatic keratinocytes can also be promoted by the microRNA, miR-17-92 (58). Together this demonstrates that several microRNAs are capable of regulating CXCL9/CXCL10 production in multiple cell types (including skin keratinocytes) and further research will be required to identify factors controlling expression of these miRNAs.

### CXCR3 in the skin

Skin tissue is composed of multiple layers that combine to form a physical barrier to infection and the external environment (59). The epidermis is a non-vascular tissue consisting of keratinocytes at different stages of differentiation, melanocytes, Merkel cells and immune cells (Langerhans cells, T cells). It is separated from the underlying dermis via a basement membrane. In contrast to the epidermis, the dermis is highly vascularized and contains lymphatic vessels and many stromal cells in addition to T cells, macrophages and dendritic cells. Epidermis and dermis can be regarded as different immunological niches as illustrated by resident memory CD8 T cells which typically reside in the epidermis and fail to recirculate to other compartments (60). Chemokine receptors such as CCR4 and CCR10, along with cutaneous lymphocyte antigen, have been associated with skin-specific homing of lymphocytes under homeostatic conditions but the induction of skin inflammation recruits additional T cells with an altered pattern of chemokine receptor expression (61). Immune effector cells such as Th1 CD4 T cells and CD8 T cells that express CXCR3 are frequently involved in inflammatory reactions and therefore it is not surprising that this receptor is often associated with inflammatory skin diseases. However, prior to inflammation, there is some evidence from CXCR3^−/−^ (and CXCL10^−/−^) mice that memory T cell accumulation within the skin may be dependent on the CXCR3/CXCL10 axis although development of tissue-resident memory CD8 T cells (Trm) in the epidermis appears to be independent of CXCR3 (62, 63). CXCR3 most likely acts not as a skin-specific chemokine receptor but instead attracts immune cells to sites of interferon-mediated inflammation (64). In a variety of inflammatory skin conditions with CXCR3^+^ T cell infiltrates, CXCL10 and 11 were shown to be produced by activated basal keratinocytes while CXCL9 was produced from dermal cells such as macrophages (65). CXCR3 and its ligands may also control the integrin-dependent adhesion of lymphocytes to the endothelial cell wall and thus control entry into inflamed skin (66). Recruitment of CXCR3^+^ cells can be enhanced by treatment of the inflammatory site with anti-IL-4 antibodies, leading to increased production of IFN-γ and CXCR3 chemokine ligands (67). IFN-β injections within the skin are also capable of inducing CXCL10/CXCR3 recruitment of T cells and macrophages while TNF-α seems to be a key inducer of CXCL10 from skin fibroblasts (68, 69).

Several inflammatory diseases serve to illustrate the role of CXCR3 in the skin. Autoimmune skin diseases such as alopecia areata (hair loss) that are associated with an IFN-γ gene signature are driven by effector T cells expressing CXCR3. Blockade of CXCR3 with antibodies prevented the development of hair loss in a mouse model of this disease (70). In the acute phase of alopecia areata, hair follicle production of CXCL10 is upregulated suggesting an involvement of this chemokine in attracting CXCR3^+^ T cells (71). In a second, chronic autoimmune skin disease, psoriasis, typically characterized by scaly, red plaques in patches on the skin, CXCL9/10/11 secretion can be induced by IL-29, produced by Th17 cells, acting on epithelial cells and melanocytes (72, 73). Consistent with the secretion of these chemokines, human psoriatic skin is also characterized by T cells, plasmacytoid dendritic cells and NK cells expressing CXCR3 (74, 75). While CXCR3^+^ cells may play a role in psoriasis, this is not the only chemokine receptor associated with disease as one study showed that intraepidermal T cells expressing CLA, CCR4 and CCR6 were more prevalent than CXCR3^+^ T cells (76). Vitiligo is an autoimmune disease that results in destruction of melanocytes and depigmentation of the skin. Active vitiligo in human patients has associated with elevated levels of CXCL10 in both serum and epidermal lesions (77). This matches with the observation of enriched CXCR3^+^, CD8 resident memory T cells in vitiligo patients (78). Patients receiving anti-PD-1 antibody therapy for metastatic tumor can also develop vitiligo-like skin lesions with infiltration of CXCR3-expressing CD8 T cells (79). Using a mouse model, Rashighi et. al. demonstrated that CXCL10-mediated recruitment, but not CXCL9-mediated recruitment, of CXCR3^+^ T cells was important for mediating this disease and that antibodies inhibiting CXCL10 might represent a viable treatment option (80). Depleting antibodies directed against CXCR3 were also able to reverse vitiligo in a mouse model (81). Widespread autoimmune diseases such as systemic lupus erythematosus [SLE] can also have skin-related manifestations that involve CXCR3 and its ligands (82–84). These studies show that the skin inflammatory infiltrate in lupus is enriched in CXCR3^+^ lymphocytes and plasmacytoid dendritic cells, the latter cell producing type I IFNs which result in CXCR3 ligand expression and amplification of the inflammatory response. Similarly, the CXCL9 and CXCL10 chemokines are highly expressed in the skin of patients with systemic sclerosis (85). A skin infiltrate of CXCR3^+^ T cells is also observed in Bullous pemphigoid, an autoimmune blistering disease, dermatomyositis, an autoimmune reaction in skin and muscle, and interface dermatitis, frequently seen in lichen planus (86–89). The common feature in many of these autoimmune skin diseases is the induction of inflammation and the subsequent production of CXCR3 ligands.

CXCR3 plays an important role in the recruitment of ovalbumin (OVA)-specific CD8 T cells into transgenic mouse skin where keratinocytes express the OVA antigen (34). Lack of CXCR3 on transgenic OVA-specific, CD8 T cells reduced skin infiltration of the T cells and the graft versus host (GVH) –like disease seen when wild-type OT-I cells were transferred. Consistent with this mouse model, cutaneous GVHD in humans is also associated with CXCR3 and its ligands in both acute and chronic forms of the disease (90, 91). Blockade of CXCR3 or CXCL11 also extends skin allograft survival in mice (92, 93). Expression of CXCR3 on non-immune cells of the skin may also play an important role in wound healing as CXCR3 deficient mice have a delayed wound healing response which can be restored by transferring CXCR3^+^ fibroblasts (94, 95). Expression of CXCR3 on keratinocytes can contribute to re-epithelisation during wound healing (96–98).

Infection of the skin frequently results in inflammation and involvement of the CXCR3 receptor. Inflammation driven by Herpes Simplex virus (HSV) contributes to the recruitment of CXCR3^+^ CD8 T cells from systemic sites and also trafficking of cells to the infected site within the skin tissue (99, 100). Clearance of epicutaneous vaccinia virus was dependent on CXCR3–expressing CD8 T cells given that transfer of wild type CD8 T cells led to viral resolution in a CXCR3^−/−^, vaccinia virus infected mouse (101). CD4^+^ skin resident memory T cells responsive to *Leishmania* are able to secrete IFN-γ and attract CXCR3^+^ T cells which aid in parasite clearance (102, 103).

Together, CXCR3 plays an important role in recruitment and function of immune cells in the skin during inflammation resulting from autoimmunity, wounds and infectious disease.

### Skin cancer and CXCR3

**Figure 1:**
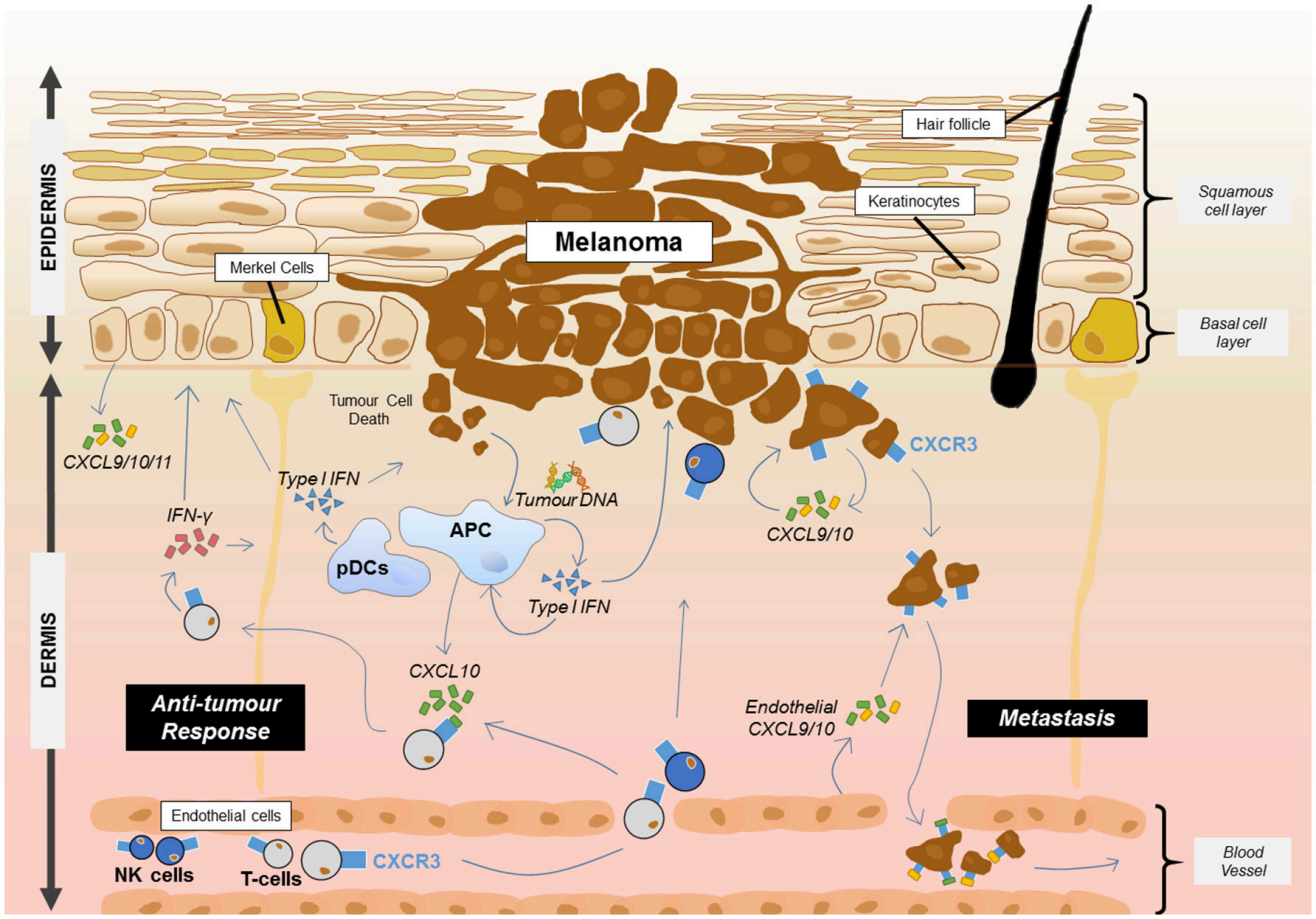
Proposed roles for CXCR3 and its ligands in melanoma. CXCR3 plays at least two key roles in melanoma. The presence of CXCR3 on melanoma cells can lead to metastasis from the primary site through endothelial cell and tumor cell production of CXCL9/10. Meanwhile, the release of DNA from melanoma cells results in uptake by APCs and the activation of the STING pathway resulting in the production of type 1 IFN. Type 1 interferon released from APCs including plasmacytoid dendritic cells (pDCs) then upregulates CXCL10 which can recruit CXCR3^+^ T cells and NK cells from the blood. Once at the tumor site, T cells and NK cells can produce IFN-γ which acts on keratinocytes, APCs and other skin cells to induce production of CXCL9/10/11 or interact with the tumour to induce cell death. This leads to further recruitment of the adaptive immune cells and anti-tumor immunity.

Skin cancers frequently arise in the epidermal layers of the skin where, most commonly, malignant transformation of melanocytes, keratinocytes or Merkel cells results from chronic exposure to ultraviolet light (104). Study of CXCR3 within this group of cancers has mainly focused on melanoma where several lines of evidence suggest that expression of CXCR3 on infiltrating T cells is associated with improved prognosis (Figure 1) (105, 106). Immunohistochemical study of primary human melanomas showed that upregulation of CXCL10 protein via type 1 interferons and the presence of CXCR3 infiltrating T cells was associated with spontaneous tumor regression (107). While it has been established that type I interferons can enhance CXCL10 expression, the source of the interferon in the tumor environment was less clear. A recent mouse study has shown that DNA derived from the tumor was able to activate the stimulator of interferon genes complex (STING) within the cytosol of dendritic cells resulting in the production of IFN-β (108). In addition, plasmacytoid dendritic cells, a key producer of type I interferons, are frequently associated with primary melanoma lesions and can be recruited to the tumor site by CCL20 (109, 110). This interferon may then act on tumor DC subsets such as the CD103^+^ DCs which have been shown to be key producers of CXCL9/10 in a mouse melanoma model and showed an association with CXCL9/10 in human disease (111). The direct contribution of primary melanoma cells to the secretion of CXCL9/10/11 is not clear although metastatic melanoma cells *in vitro* can produce CXCL9/10/11 in response to IFN (112). One human study showed that a higher frequency of metastatic melanoma samples expressed the CXCL10 gene relative to primary melanoma samples (113). In a therapeutic setting for melanoma, adjuvant IFN-α therapy of melanomas is known to upregulate CXCL10 production while CXCL9 and CXCL10 were induced in melanomas by chemotherapy agents such as cisplatin (114). Intralesional BCG can also increase production of CXCL9/10/11 which promotes γδ T cell recruitment and regression of melanomas (115). One melanoma study suggested that under IFN-γ stress, melanoma variants which fail to produce CXCL9 could be generated as a mechanism of immune escape (116). In addition to T cells, CXCL10 can also promote the trafficking of adoptively transferred NK cells into melanoma where they cause regression of the tumor mass (117).

While CXCR3 expression on infiltrating immune cells generally restrains the melanoma, the expression of this receptor on melanoma cells themselves can lead to metastasis (118, 119). In mouse studies with B16F10 cells, reduction of CXCR3 expression within tumor cells by anti-sense RNA resulted in less frequent metastasis (120). Metastasis can be promoted by endothelial cell secretion of CXCL9 (and CXCL10), assisted by VEGF, within the tumor microenvironment or autocrine CXCL10/CXCR3 interactions on tumor cells (113, 121, 122). Consequently, metastasis represents a situation in which blocking the CXCR3 receptor on tumor cells might be beneficial. In this regard, the design of small molecule antagonists of CXCR3 should be beneficial, particularly if precisely targeted to the tumor cells (123).

Melanoma represents one situation in which metastasis moves cancer cells away from the skin but we must also consider a role of CXCR3 in attracting cancer cells to the skin. Cutaneous T cell lymphoma is an unusual situation where CXCR3 expression on malignant T cells helps recruit and establish this cancer within the skin (124, 125). In this scenario, CXCR3 has been associated with epidermal-trophic cutaneous T lymphomas rather than dermal residing tumors suggesting that CXCR3 ligands may be expressed from the epidermis in this disease setting (126). Interestingly, while T cell lymphomas may use CXCR3 to initially migrate to the skin, the presence of high levels of CXCR3 ligands in the blood, at least partly due to secretion by the lymphoma cells, leads to downregulation of CXCR3 on effector CD8 T cells such that they do not accumulate in the skin (127, 128). This represents a novel form of immune escape in advanced cutaneous T cell lymphoma. Additional cutaneous lymphomas/leukaemias have also reported expression of the CXCR3 receptor on tumor cells including in epidermotrophic B cell lymphoma, lymphomatoid papulosis and skin lesions of leukemic plasmacytoid dendritic cell neoplasms (129–131).

Less well understood is the role of CXCR3 and its ligands in non-melanoma skin cancers such as squamous cell carcinoma (SCC) and basal cell carcinoma (BCC). The lack of studies in this area may reflect the relatively benign nature of many cutaneous BCC/SCCs which are generally removed through surgical procedures (132). However, a subset of SCCs can be metastatic with an estimate of 0.025–20% of premalignant actinic keratosis (AK) lesions progressing to invasive SCC (133). Immunocompromised patients (particularly those undergoing solid organ transplantation or HIV patients) have a 65- to 250-fold increased risk of developing SCC suggesting that immune responses play a role in controlling these tumors (134, 135). Gene profiling of BCC and SCC tumor tissue suggests an increase in IFN related genes including CXCL9 while immunohistochemical staining demonstrated the presence of CXCR3^+^ immune cells (136). Imiquimod is a clinically approved, topical treatment for SCC/BCC and can induce type 1 IFN signaling through interaction with TLR7 leading to the downstream recruitment of CXCR3^+^ T cells (137). The expression of CXCL 10/11 and CXCR3 has also been demonstrated in human keratinocytes derived from BCCs (138). In addition, CXCL11 is capable of promoting immunosuppressive indoleamine 2,3-dioxygenase (IDO) expression in human basal cell carcinoma and enhancing keratinocyte proliferation, thus potentially reducing the anti-tumor activity of any infiltrating CXCR3^+^ effector T cells (138, 139). Consequently, it is still unclear whether the CXCR3 immune infiltrate in human SCC and BCC is associated with tumor regression or progression. In a mouse model of skin epithelial carcinogenesis promoted by DMBA/TPA, gene deletion of CXCR3 produced a lower incidence of skin tumors (140). Both CXCR3-expressing CD4 and CD8 T cells were seen to infiltrate the skin and promoted keratinocyte proliferation (140). The contribution of CXCR3 to tumor development in this mouse model would be consistent with a known role for inflammation in promoting DMBA/TPA tumors (141).

In contrast to this tumor model, we have recently demonstrated that CXCR3 and associated chemokine ligands are important in attracting an effector T cell population to the hyperplastic ear skin of mice transgenic for the human papillomavirus (HPV16) E7 oncogene (142). In this mouse model, HPV16E7 protein is expressed in epithelial cells under the control of a keratin 14 promoter. Intracellular binding of E7 to the retinoblastoma (Rb) protein leads to a dysregulated cell cycle within keratinocytes and the subsequent development of a precancerous, hyperplastic epithelium resembling actinic keratosis, a precursor lesion which can progress to squamous cell carcinoma in human patients (143). One feature of the E7-driven hyperplasia is a chronic inflammatory/wound healing microenvironment associated with elevated levels of IFN-γ and immune cell infiltration (144, 145). Chronic IFN-γ production in the skin, from cells such as infiltrating NKT cells, results in an immunosuppressed microenvironment via mediators such as IDO (146–148). The presence of IFN-γ was also shown to induce CXCL9 and 10 production from CD45^−^ cells in the epidermis and a resulting infiltrate of CXCR3^+^ T cells (142). These CXCR3^+^ T cells were shown to be effector cells mediating skin graft rejection in experiments where the E7-expressing graft was devoid of suppressor lymphocytes. Opposing outcomes for the role of CXCR3^+^ T cells between the HPVE7 transgenic mice and the DMBA/TPA treated mice suggests the need for SCC models which more closely mimic UV-induced cancer in humans to resolve the clinical benefit of attracting CXCR3^+^ T cells as an immunotherapy. Applying strategies that reduce local immunosuppression will also be important in SCC/BCC.

## Concluding remarks

Within the skin, CXCR3 and associated chemokine ligands form a very important chemotactic response to interferon-mediated inflammation. Recruitment of CXCR3^+^ immune cells can aid in the response to skin infection while also enhancing autoimmune conditions such as psoriasis. The role of CXCR3 is more complex in skin tumors where expression of this chemokine receptor on tumor cells can assist in tumor metastasis or the suppressive microenvironment of the tumor can overcome recruited CXCR3^+^ effector T cells. In addition, it is possible that skin CXCR3^+^ T cells may promote inflammation-driven cancer development in some instances. Consequently, the skin tumor type and a better understanding of the regulation of expression of human CXCR3 isoforms on skin tumor cells and their differential responses to CXCL9/10/11 will dictate if this chemokine receptor/chemokine system can be manipulated to treat skin cancers. Blockade of CXCR3 on tumor cells, thus preventing metastasis, combined with increased skin expression of CXCL9/10/11 might be appropriate in circumstances where CXCR3^+^ effector T cells can reduce tumor growth. Changes in the expression of CXCR3 and its ligands may be achieved through the manipulation of host miRNAs, an approach that warrants further investigation in animal models. Recruited effector T cells must also overcome the local suppressive environment generated by skin tumors and therefore a combination of chemokine attraction using CXCR3 ligands and modulation of T cell checkpoint molecules (e.g., PD-1) or IDO may be necessary to promote tumor regression.

## Author contributions

All authors listed have made a substantial, direct and intellectual contribution to the work, and approved it for publication.

### Conflict of interest statement

The authors declare that the research was conducted in the absence of any commercial or financial relationships that could be construed as a potential conflict of interest.
